# Supplementary material for: Physiological adaptations and myocellular stress in short-term, high-frequency blood flow restriction training: A scoping review
Source: PLoS One. 2022 Dec 30;17(12):e0279811. doi: 10.1371/journal.pone.0279811 (PMC9803189; doi:10.1371/journal.pone.0279811)
Supplement: Attachment — Submitted filename: RESPONSE TO REVIEWERS.docx [file pone.0279811.s003.docx]

**RESPONSE TO REVIEWERS**

**REVIEWER 1**

Given the emphasis on myocellular stress, a figure collating some of the data on satellite cells, muscle damage, inflammation would add novelty to the manuscript. The provided figures largely reflect muscle size and strength and have been shown many times before. If a summary figure is difficult to tabulate given the limited studies directly assessing myocellular stress, perhaps a graphical abstract of sorts could be created linking some of the ideas proposed by reviewers. Acute inflammation can activate satellite cells, promoting myonuclear accrual, etc. and how BFR specifically can initiate these signaling cascades. This would be a great reference tool for researchers and clinicians seeking to understand or explain potential risks of high frequency BFR.

**Response:** We added a figure to our discussion. Introducing the relationship between muscle damage and satellite cell activation. We hope we have responded to the reviewer's request.

**REVIEWER 2**

**Introduction:**

**Lines 53-54:** “In addition, low-intensity aerobic training programs with BFR can promote muscle hypertrophy, increases in lower limb strength, and aerobic capacity despite low intensities of training [3,4].” - - - Is this relative to a non-BFR aerobic control?

**Response:** Perfect! Good observation. Yes, this is relative to a non-BFR aerobic control. We emphasize this aspect in this new version (Lines 56-57). Thank you very much!

**Intro, paragraph 2:** In general, this paragraph seems to lack focus. Some ideas on frequency are presented…but I believe it can be developed some. For example, authors reference powerlifters at the end, but what point is being made with this inclusion? “…appears to maximize hypertrophic adaptions in well trained powerlifting athletes” demonstrating that the addition of BFR may provide…

**Response:** Perfect! Good observation. In this paragraph, we seek to present high-frequency and short-term blood flow restriction training and its possible applicability. We agree that the paragraph could be improved. As requested by the reviewer, we have added additional information regarding the applicability of high-frequency blood flow restriction training for highly trained athletes (Lines 67-69). We hope we have complied with the reviewer's request. Thank you very much!

**Intro, paragraph 2:** “High-frequency BFR training can be a potentially useful tool to accelerate recovery in clinical rehabilitation settings, since the technique can provide positive physiological adaptations in less time compared to high-load strength training [6].” - - If it is high frequency is it really less time? Or is this compared to a high load training program of similar training frequency?

**Response:** The statement concerns the capacity of high-frequency training with blood flow restriction to promote short-term adaptations.

In it’s current form the introduction does not provide a compelling rationale for the present manuscript. For example authors primarily provide evidence from Landow and Nielson as examples of discrepant findings in strength outcomes between high frequency BFR programs. However, I would argue that this is true in the majority of the BFR literature regardless of frequency. Increases in 1RM strength are generally inconsistent across the BFR literature. However, when performed with low loads, BFR will typically provide similar changes in muscle size. It would be more compelling to provide muscle growth data (if available).

**Response:** Perfect! We added information related to the differences presented about the muscle fiber size (Line 82-84). However, we believe that it is important to provide information related to strength tests, especially because we present evidence of strength decline after high-frequency blood flow restriction training.

Discussion:

**Lines 520-522:** “Regardless of exercise mode, sessions per week (> 4), and method of assessment (e.g., maximal isometric voluntary contraction), it appears that HF-BFR training generally produces superior increases in muscle strength when compared to the same intensity exercise performed without BFR.” – What do authors mean by “same intensity” Some would suggest this would refer to the proximity to failure. I believe that the authors are referring to same “relative training load (%1RM or same RM). However, it would be important to specify both proximity to failure and %load/RM.

**Response:** Great! In this new version, we emphasize that the intensity concerns the external load (Line 533). Thank you very much!

**Lines 530-532:** “Intriguingly, the results appear to indicate that HF-BFR application may produce similar gains in muscle strength as longer duration protocols (> 3 weeks) in less time despite differences in exercise mode, population studied and BFR application parameters. - - Given the large range in strength changes reported, I am not sure this is as clear as the authors suggest. Again, I would encourage authors to consider the current consensus on strength adaptations to low load alternatives of training. They are typically difficult to predict and typically underperform compared to high load alternatives. Authors indicate that strength outperformed the comparison group 78% of the time….however what that “comparison group” was is really important for the interpretation of what this means.

**Response:** Perfect! In this new version, we specify that the information presented refers to comparisons between BFR low-intensity versus non-BFR low-intensity. In addition, we present results regarding BFR low-load versus non-BFR high-load comparisons.

Not much discussion of provided on muscle size other than a comparison to longer duration values. However, this is (as the authors mention) not the purpose of the present manuscript. It may be more important to discuss why some had larger and others had smaller changes in muscle size. It may also be worthwhile to discuss differences in control conditions across studies. For example, it appears that many of the control groups were volume matched. This may simply indicate the control groups were underdosed…as low loads performed to failure typically produce similar growth as low loads with BFR to failure (although BFR conditions will most typically perform less volume). If there was growth what does this indicate? What are the implications of this finding?

I think one issue with the present interpretation is that no study (unless I missed it) compared low/regular frequency BFR over the same period of time (i.e., 3-4 weeks) to the high frequency BFR condition. Thus, it is unclear if there really is a benefit of the increased frequency compared to more common frequencies of training. This is found later in the limitations section…but it does make it difficult to recommend high frequency BFR training when it is unknown if it is better than a lower frequency option.

After re-reading this manuscript it seems to outline (through the limitations sections) barriers and issues with interpreting the high frequency BFR literature when considering its incorporation into clinical practice. However, the introduction provides a different expectation. I would work on overall flow to increase the cohesiveness of this manuscript. I would recommend expanding the discussion on the findings of this investigation while reducing focus on limitations. I may be wrong on this recommendation and encourage the authors to consider all feedback along with your own thoughts/opinion.

**Response:** We understand the reviewer's concern, however, we should point out that we are dealing with a scoping review. In accordance with the recommendations for scoping review, the purpose of the discussion is to present the main results and discuss limitations and guide future research efforts. In our conception, we believe we have complied with this proposal. We would like to point out that our focus was to provide an overview of training with high-frequency blood flow restriction, including the limitations of the studies that analyzed this training methodology so that future research could be better equipped to conduct a rigorous trial and avoid the pitfalls of the past prescriptions. We have added a number of references to the importance of discussing limitations, as well as a supportive reference (12).
