# Supplementary material for: Physiological adaptations and myocellular stress in short-term, high-frequency blood flow restriction training: A scoping review
Source: PLoS One. 2022 Dec 30;17(12):e0279811. doi: 10.1371/journal.pone.0279811 (PMC9803189; doi:10.1371/journal.pone.0279811)
Supplement: Attachment — Submitted filename: RESPONSE TO REVIEWERS.docx [file pone.0279811.s004.docx]

**RESPONSE TO REVIEWERS**

**REVIEWER 2**

1. Introduction:

I found the introduction to be much improved on the latest revision. Thank you for the changes that have been made.

**Response:** Thanks for the help provided!

2. Results:

MVC section line 243: Are authors referencing exclusively low load training controls without BFR? “All studies included analyses of the isometric strength of the knee extensors and only one did not identify a significant increase after BFR training [24], while no study identified increases in isometric strength after non-BFR training.” - -∫I would add an additional descriptor here.

**Response:** Perfect! We emphasize that we are referring to low load/intensity training. Thank you very much! (Line: 246)

3. Throughout the results authors are sometimes specific what the control group is and other times will refer to as a control.

**Response:** In this new version, we adopted only "comparator". Thanks for the comment.

4. Muscle endurance results section: Were “differences between groups washed out after 12 weeks despite continued training? After 4 weeks post-surgery, the high-frequency BFR training group exhibited ~50% greater sub-maximal quadriceps endurance than the non-BFR work matched control group, but the differences between groups washed out after 12 weeks.

**Response:** Thanks for the comment. As specified, BFR training was applied before surgery. After surgery, participants were involved in a rehabilitation program (without BFR training). We point out this aspect in this new version. (Line: 279-280)

5. Jump and sprint section: BFR produced superior decreases relative to what?

“The results of this study indicate BFR produced superior decreases in 30-m sprint time that was attributed to improvements in early acceleration during the first 10-m.”

**Response:** Good observation! We refer to low-load training without BFR. We highlight this point in this new version. Thank you very much! (Line: 290-291).

6. Muscle size lines 307-309: Was the control a low load intervention? “From the 2nd to the 14th post-surgery day, a group of patients underwent an BFR exercise intervention, while a control group underwent an exercise intervention without BFR.”

**Response:** Good observation! The study in question analyzed exercise without external overload. We highlight this aspect in this new version. Thank you very much! (Line: 312-313).

7. Microscopic measurements section lines 351-353: …in the control condition which performed…

blocks of high-frequency BFR training versus heavy load training without BFR in a traditional high load training routine; in the BFR condition, there was a significant increase in CSA of type I fibers, butnot of type II fibers; in the control condition, no significant differences were reported.

**Response:** Perfect! We made the change suggested by the reviewer! Thank you very much! (Line: 360)

8. Inflammatory responses lines 473-474: Was this low load? I would add low load after high frequency. “One study [28] analyzed macrophage content by immunofluorescence before, after eight days, and three and ten days after three weeks of high-frequency training with and without BFR.”

**Response:** Good observation! We point out that the intervention tested low-load training. (Line: 485).

9. Vascular adaptations lines 496-497: Was this low load? One study [8] analyzed the number of capillaries per muscle fiber and changes in the perivascular basement membrane in high-frequency training with and without BFR.

**Response:** Good observation! We point out that the intervention tested low-load training. (Line: 509).

10. It may seem redundant to the authors but it may be most appropriate to add “low load” whenever discussing “resistance training with BFR” as this provides further detail which may be important to the reader.

**Response:** Perfect! When necessary, we add “low-load” to discussions around resistance training with BFR. Changes are highlighted throughout the text.

11. Discussion lines 570-573: How was edema measured? “Their results seemed to indicate that the hypertrophic response to BFR occurred without the presence of edema (in 3 weeks) whereas in high load training, concomitant edema occurs (at week 3), impairing the initial adaptation process yet over the same number of sessions, tended to induce greater muscle hypertrophy and muscle strength (at 6 weeks).”

**Response:** Good observation. We present the method used in the study to identify edema. Thank you very much! (Line: 580-581)

12. Limitations section lines 623-625: Many studies have actually employed 40% AOP…so 40 may be more appropriate than 50% - - “Research has shown that there appears to be a minimal threshold of applied pressure (50% of arterial occlusion pressure, AOP) needed to accelerate fatigue accumulation during BFR training [35],..”

**Response:** Good observation. However, the information presented is supported by the cited article. In this sense, we chose to keep it at 50%.
